# Progress in the synthesis of δ-sultones

**DOI:** 10.1007/s00706-017-2010-0

**Published:** 2017-07-15

**Authors:** Christina Gaunersdorfer, Mario Waser

**Affiliations:** Institute of Organic Chemistry, Johannes Kepler University Linz, Altenbergerstraße 69, 4040 Linz, Austria

**Keywords:** Heterocycles, Sulfonates, Cyclization reactions, Transition metal catalysis

## Abstract

**Abstract:**

Sultones, the cyclic esters of hydroxyl sulfonic acids, are a fascinating class of heterocycles and the recent years have witnessed an increasing interest in these molecules, especially in six-ring δ-sultones. The importance of these compounds is either because of their biological properties themselves or due to their versatility as intermediates in more complex target syntheses. Accordingly, the development of new synthesis methods to access δ-sultones is an important and rewarding task which we wish to highlight in this review.

**Graphical abstract:**

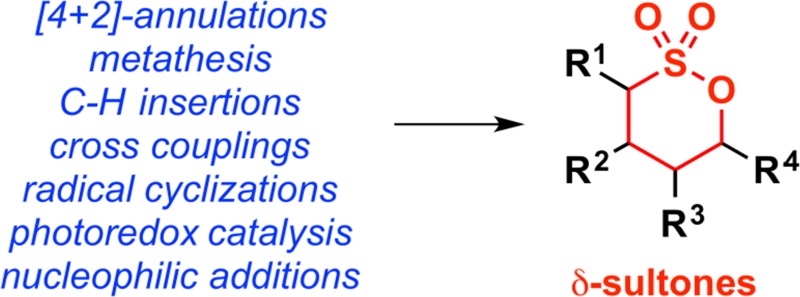

## Introduction

In 1888, Erdmann first introduced the term “sultone” to describe the cyclic esters of hydroxyl sulfonic acids [1]. Over the decades, these compounds have received considerable attention (for some earlier general reviews, please see [2–6]). The interest in these heterocycles is either due to their biological properties themselves (please refer to [7–13] for some selected case studies of sultones in medicinal chemistry) or because of their versatility as intermediates for further transformations, such as, e.g., sulfoalkylations or desulfurizations, to mention a few examples only (which is very nicely discussed in a recent review by Mondal [6]). Like lactones, these sulfur analogues can be classified according to their ring size as β-, γ-, δ-, or ε-sultones. The syntheses of these important targets, either in a racemic or in an asymmetric fashion, were found to be a challenging task. Accordingly, it comes as no surprise that the development of new strategies to access sultones became an important research topic. Interestingly, the first reviews dealing with the syntheses of sultones date back to the 1950s already [2], illustrating the longstanding importance of these targets. In contrast to the syntheses of lactones from the corresponding hydroxycarboxylic acids by classical esterification methods, sultones can in general not be accessed by such strategies [2, 14–19]. The early synthesis approaches mainly relied on the direct distillation of halo- or hydroxysulfonic acids under vacuum [14–17] to give 5–7 membered-ring sultones or on sulfonation reactions of alkenes with SO_3_ (for pioneering reports by Bordwell et al., see [18, 19]) (Scheme 1). The later method even allows for the synthesis of highly reactive β-sultones, and over the years, these methods have been significantly developed further [2–6]. Among the differently ring-sized sultones, δ-sultones have received considerable attention over the last two decades. This recent interest is on the one hand because of the importance of these targets in medicinal chemistry either as such or as synthesis intermediates. On the other hand, a lot of groups focusing on the development of new synthesis and catalysis methodologies successfully demonstrated the potential of their new methods to facilitate the synthesis of δ-sultones. Accordingly, a broad variety of new concepts to access these important targets either in a racemic or in an asymmetric fashion have been introduced. Because of some very powerful recent developments, we herein wish to highlight the most important strategies to access these targets with a special focus on the developments of the last 2–3 decades (for a detailed overview of earlier studies, please see the very illustrative review by Roberts and Williams [4]).

**Figure:**
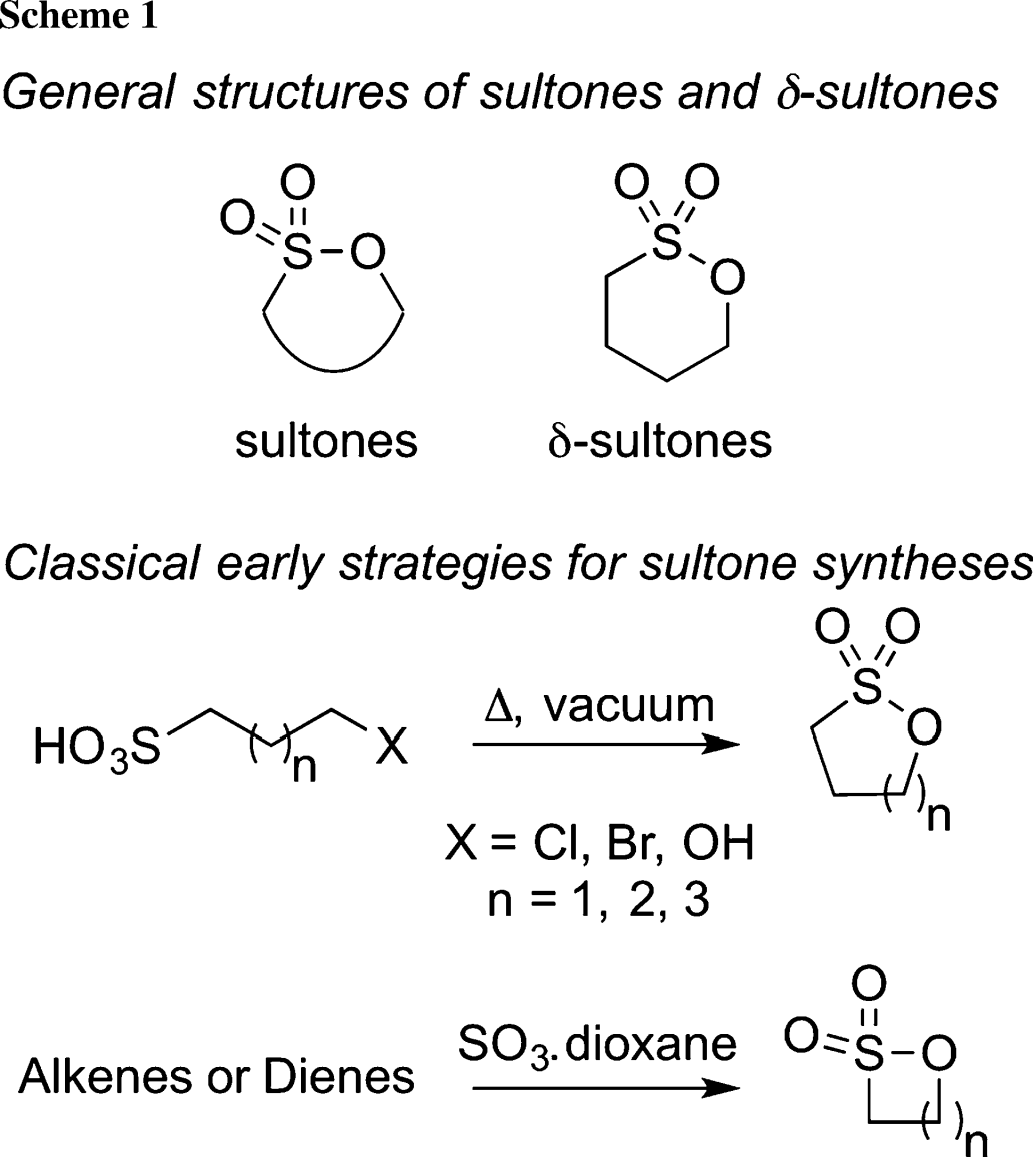

## [4+2] Annulations

In general, the syntheses of six-membered-ring compounds via [4+2]-type cyclization approaches (i.e., Diels–Alder reactions) are a very well and heavily investigated topic (for an illustrative general overview, please see [20]). The versatility of vinyl sulfonates as highly reactive dienophiles has been known for decades [21], and thus, it comes as no surprise that Diels–Alder reactions have emerged as a powerful strategy to access δ-sultones [22–41]. In 1989 already, the group of Metz reported the intramolecular Diels–Alder reaction of furan-based vinylsulfonic acid esters **3** to give the tricyclic δ-sultones **4** in high yields and with full stereo-control (Scheme 2) [22]. Very importantly, these reactions proceed more or less spontaneously at room temperature already, making this Diels–Alder strategy a very powerful and straightforward approach to access these δ-sultones from simple furan-based alcohols, such as compound **1** and vinylsulfonic acid chloride (**2**). Since that pioneering report, this protocol and subsequent improvements and developments have been amongst the most important strategies to access polycyclic δ-sultones, which then served as versatile intermediates for numerous further transformations [23–35]. For example, in 1994, Metz and co-workers reported the synthesis of the sultone **6** via a Diels–Alder strategy starting again from 2-acetylfuran (**5**). This compound was then successfully further transformed into the 1,10-*seco*-eudesmanolides ivangulin (**7**), eriolanin (**8**), and eriolangin (**9**) [23–26] (Scheme 2, lower part).

**Figure:**
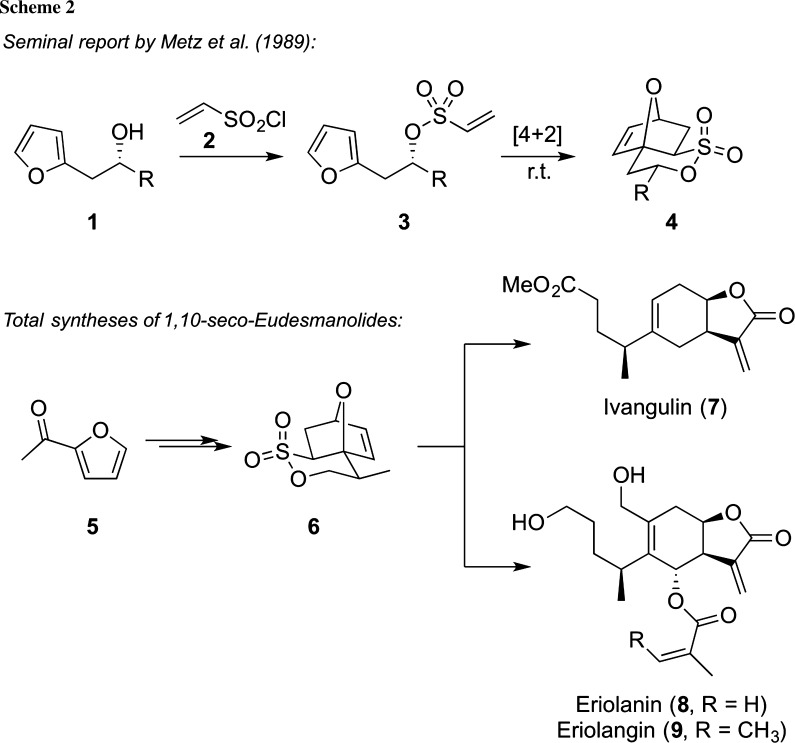

One particularly impressive example that illustratively highlights the potential of this powerful sultone chemistry is the total synthesis of pamamycin-607 (**14**), again by Metz’s group [28–32] (Scheme 3). Hereby, the northern and southern fragments were successfully accessed via a sultone-forming Diels–Alder approach starting from furans **10** and **11** which, upon reaction with vinylsulfonyl chloride, directly resulted in the highly decorated δ-sultones **12** and **13**. These advanced complex intermediates were then used further to access pamamycin-607 (**14**) in an elegant and efficient manner. It is also impressive to see that this strategy was successfully applied later on to synthesize other structurally related chiral tetrahydrofuran-containing complex target molecules from bicyclic δ-sultones intermediates, such as compounds **12** or **13** [33–35].

**Figure:**
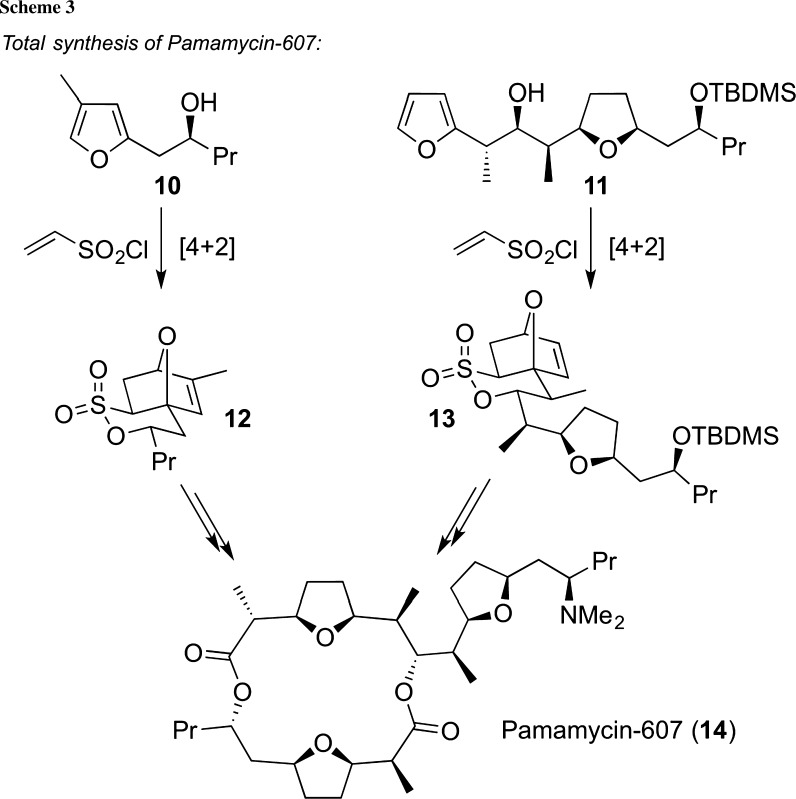

Besides furans, also other commonly [4+2]-cyclization-established electron-rich dienes, such as, e.g., cyclopentadiene **15** or acyclic dienes **21,** have been equally successfully utilized for such δ-sultone-forming intramolecular Diels–Alder approaches (please see the upper reactions in Scheme 4 for general case studies) [36–41]. As illustrated in the lower example in Scheme 4, the resulting δ-sultone products were then again very successfully applied for different demanding further applications, such as, for example, the total synthesis of (−)-myltaylenol (**26**) reported by Winterfeldt et al. [40, 41].

**Figure:**
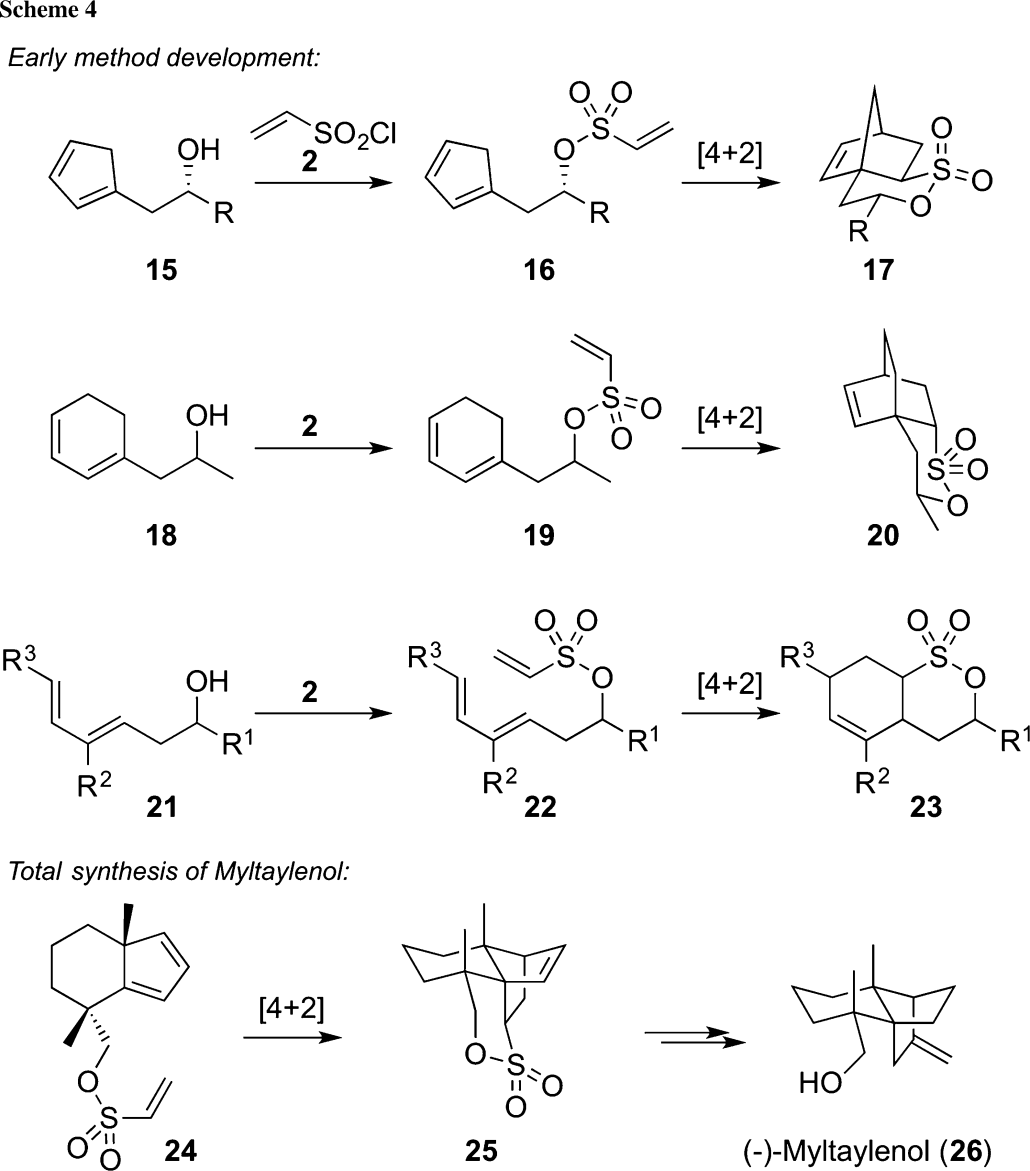

Although Diels–Alder approaches are by far the most important and prominent [4+2]-cyclization reactions, it is fair to say that over the years, also other strategies to access six-membered-ring carbo- or heterocycles have been introduced, especially when it comes to asymmetric transformations. For example, the use of the so-called type 1 ammonium enolates, usually generated from ketenes or acylhalides in the presence of a chiral nucleophilic catalyst, has resulted in a powerful methodology to generate differently ring-sized compounds upon reaction with a variety of acceptors (for a very illustrative overview, please see [42]). In analogy, Peters and co-workers succeeded in introducing a highly enantioselective [2+2]-cyclization protocol for the syntheses of chiral β-sultones and β-sultams starting from simple sulfonyl chlorides **27** [43, 44]. The reactions are supposed to proceed via the corresponding chiral zwitterionic intermediates **28** (obtained by the addition of the catalyst to an in situ formed sulfene), which undergo cyclizations in close analogy to classical ketene-based type 1 enolates. Our group has for years been interested in reactions that proceed via chiral ammonium enolates (for recent examples, please refer to [45–47]) and we thus rationalized that this elegant strategy should also be extendable to δ-sultone syntheses (Scheme 5, lower reaction, unpublished results). Although we were able to obtain a first proof-of-principle for this reaction, we were not able to carry it out in a high yield on a broad substrate scope and also the use of chiral amines, such as, e.g., Cinchona alkaloids did not give any products **31**, thus making this strategy of rather limited applicability only and clearly less practical than the analogous β-sultone protocol developed by Peters et al.

**Figure:**
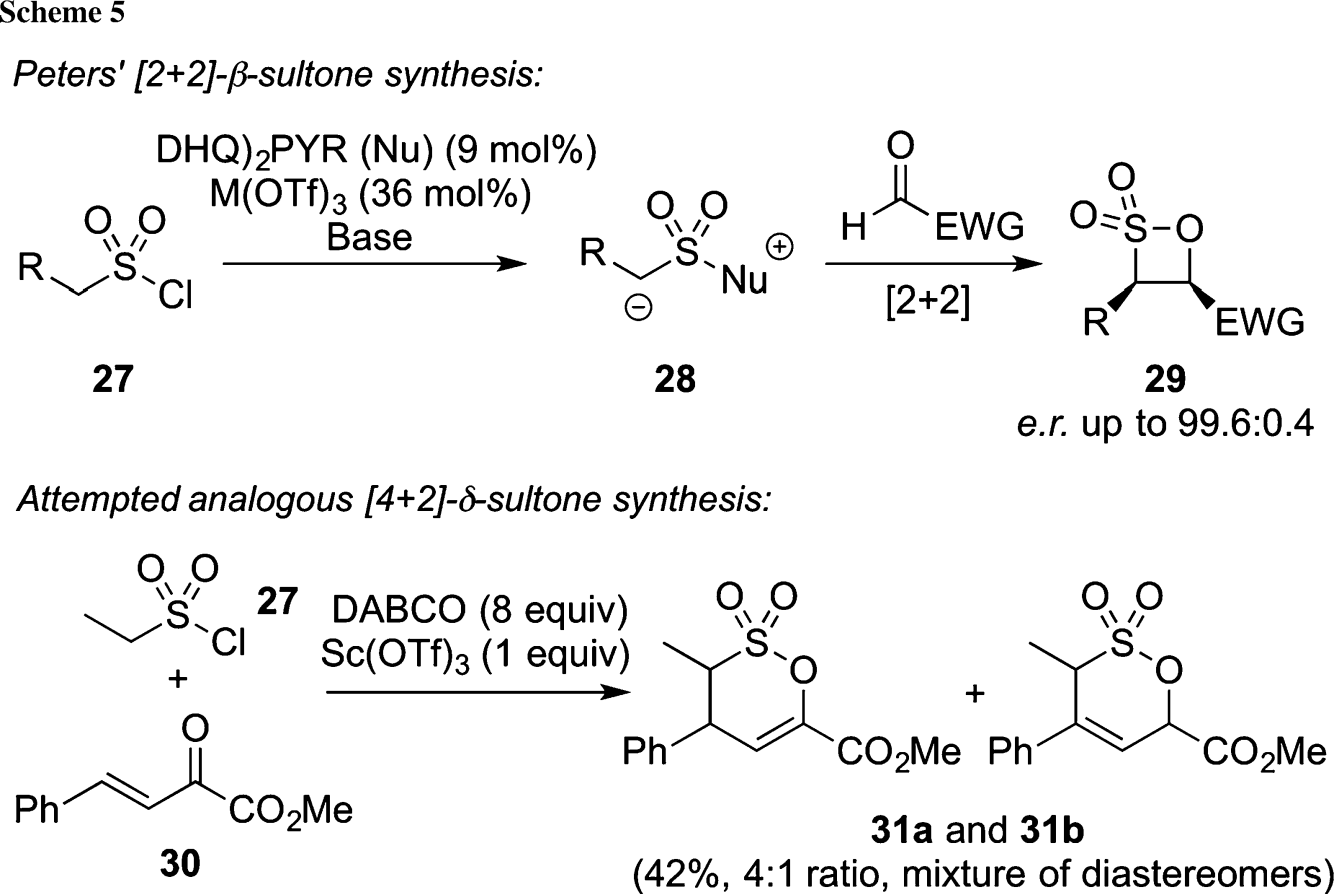

## [3+3] Annulations

A powerful alternative strategy to carry out enantioselective annulations is the use of *N*-heterocyclic carbenes (NHCs) as catalysts. Depending on the used starting materials, this strategy allows for six-ring formations either via [4+2]-type cyclizations or also [3+3]-type annulations [48, 49]. The Lupton group has recently succeeded in expanding this versatile concept to the synthesis of δ-sultones **35** starting from simple α,β-unsaturated sulfonyl fluorides **32** [50]. This starting material gives the corresponding sulfonyl azolium intermediate **33** upon reaction with an NHC catalyst. This compound can then react with 1,3-dinucleophiles, such as TMS-protected enolethers **34** in a [3+3] cyclization to give the corresponding δ-sultones **35** in a highly efficient manner. It was also shown that the use of a chiral NHC catalyst allows for an asymmetric reaction, albeit so far with moderate enantioselectivities only [50] (see Scheme 6).

**Figure:**
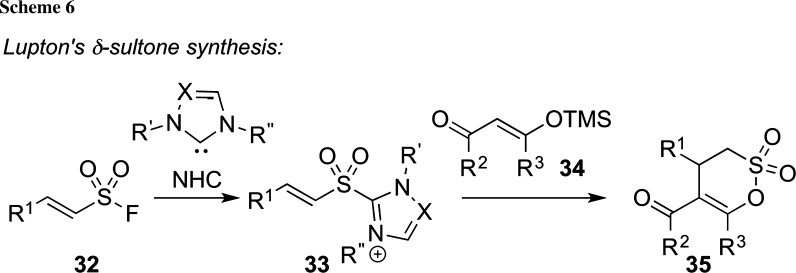

## Metathesis approaches

The versatility of alkene and alkyne metathesis reactions for the (organic) chemistry community cannot be overestimated and this importance was very impressively highlighted by the 2005 Nobel Prize in chemistry awarded to Yves Chauvin, Robert Grubbs, and Richard Schrock [51–53]. Among the different metathesis approaches developed so far, ring-closing metathesis reactions have emerged as a powerful tool in complex total syntheses and other synthesis fields as they represent one of the most important and also atom-efficient protocols to form differently ring-sized carbo- and heterocycles [54]. Therefore, it comes as no surprise that ring-closing alkene metathesis has also served as a valuable strategy to access sultones from simple olefinic precursors. Pioneering work in this field again comes from Metz et al., who described an elegant synthesis of five- to nine-membered-ring size sultones in 2002 already (Scheme 7) [55, 56]. As so often in metathesis reactions, Grubbs’ second generation ruthenium-based catalyst **37** turned out to be the catalyst of choice for this transformation, giving access to a variety of different sultones **38** (among them the corresponding δ-sultone). In 2014, Brückner et al. then used an analogous approach to access the sultone **40**, which can be considered as a sultone-protected homoallylic alcohol which served as a versatile building block in the synthesis of a key-fragment of the unnatural enantiomers of different polyene polyol antibiotics [57].

**Figure:**
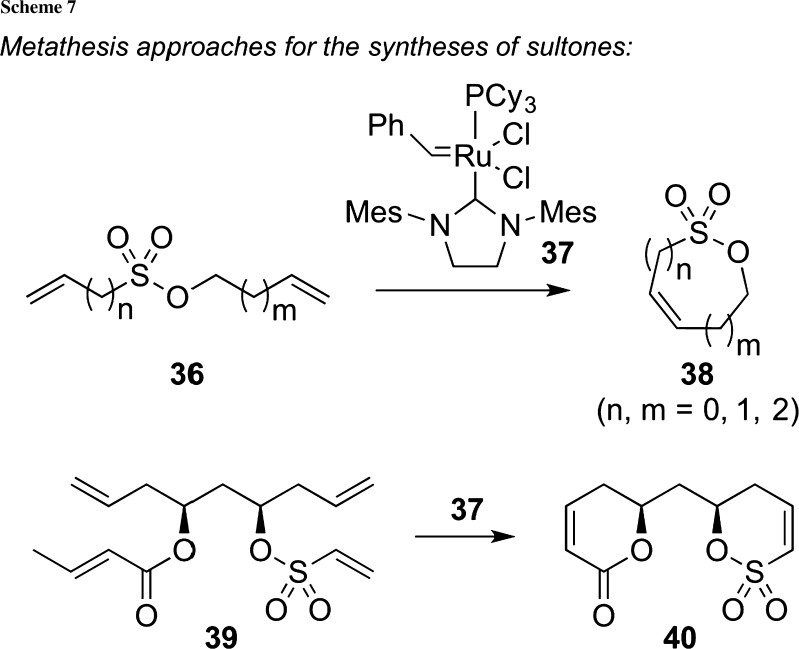

## Rhodium-catalyzed C–H insertion reactions

The catalytic activation of non- or only weakly-polarized C–H bonds is a very important but also rather difficult target. Accordingly, investigations focusing on the introduction of efficient C–H insertion reactions have become one of the hottest topics over the course of the last years. Gratifyingly, the recent developments of new methods for C–H insertion reactions of metal carbenoids or nitrenoids have led to numerous highly spectacular transformations that are otherwise only very difficult to achieve and have, therefore, led to a completely new retrosynthetic thinking [58]. In 2007, the groups of Novikov [59] and Du Bois [60] independently reported Rh-catalyzed strategies for the synthesis of ester-containing δ-sultones, such as compounds **42** and **46** (Scheme 8). While Novikov mainly relied on α-diazoesters **41** to generate the corresponding reactive Rh-carbene species, Du Bois showed that besides α-diazoesters also in situ generated aryliodonium ylides (obtained by treatment of **45** with PhIO in the presence of a base) can serve as Rh-carbenoid precursors, thus leading to a versatile strategy to access highly functionalized δ-sultones from simple (chiral) starting materials. The potential of the hereby obtained target compounds to serve as intermediates for further transformations was also well document [59–64]. For example, sultones **42** can be transformed into lactones **43** upon treatment with SmI_2_ [61], while the citronellol-based chiral sultone **46** could be used in the total synthesis of bakuchiol (**47**) as shown by Novikov’s group [63].

**Figure:**
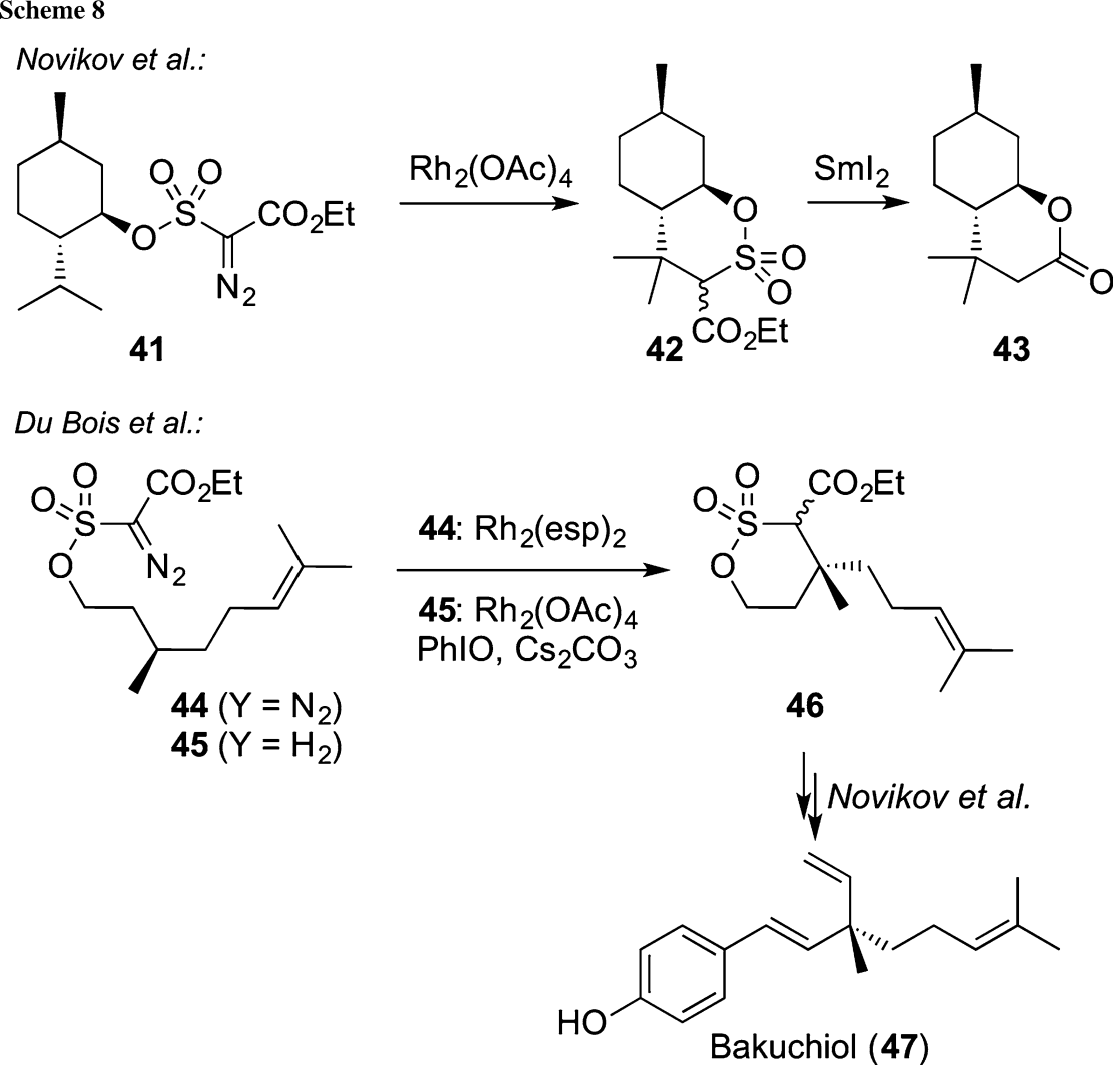

## Palladium-catalyzed cross-coupling reactions

The introduction and systematic development of palladium-catalyzed cross-coupling reactions over the course of the last decades has changed the strategic and retrosynthetic thinking of chemists like no other modern methodology in the field, and the 2010 Nobel prize in chemistry awarded to Richard Heck, Ei-ichi Negishi, and Akira Suzuki is just one of the numerous prestigious awards highlighting the importance of this topic [65–67]. In 2009, Majumdar and co-workers reported the palladium-catalyzed intramolecular Heck-type cyclization of easily accessible bromine-containing sulfonic acid esters **48** [68] (Scheme 9). This efficient protocol provides a straightforward access of polycyclic sultones **49** and, depending on the used cyclization precursors, can also be applied to larger ring systems [69] or heteroaryl-containing starting materials [70]. In 2012, Doucet’s group then reported a further improvement of this methodology with respect to catalyst loading and application scope, thus making these intramolecular arylation approaches highly versatile and functional group tolerant to access a broad variety of such polycyclic δ-sultones [71]. Besides using halide-based aryl starting materials for cross-coupling cyclization reactions, the direct oxidative Pd-catalyzed coupling of C(sp^2^)-H bonds in the presence of an oxidant has also been successfully applied to the synthesis of δ-sultones and sultames [72–74]. For example, Laha et al. described the Pd(OAc)_2_-catalyzed intramolecular coupling of sulfonic acid esters **50** in the presence of an excess of AgOAc to access sultones **49** and the corresponding sultames [74]. One particularly impressive case study that substantiates the potential of such oxidative Pd-catalyzed cyclization reactions was reported in 2012 by Wang’s group. Using Pd(OAc)_2_ and K_2_S_2_O_8_ as an oxidant, they succeeded in carrying out an intermolecular sultone formation between [60] fullerene (**51**) and simple arylsulfonic acids **52** (Scheme 9, lower reaction) [73]. Worth to note, besides using fullerene coupling partners, this strategy can also be successfully applied to other aromatic compounds as demonstrated by the same group in 2012 already [72].

**Figure:**
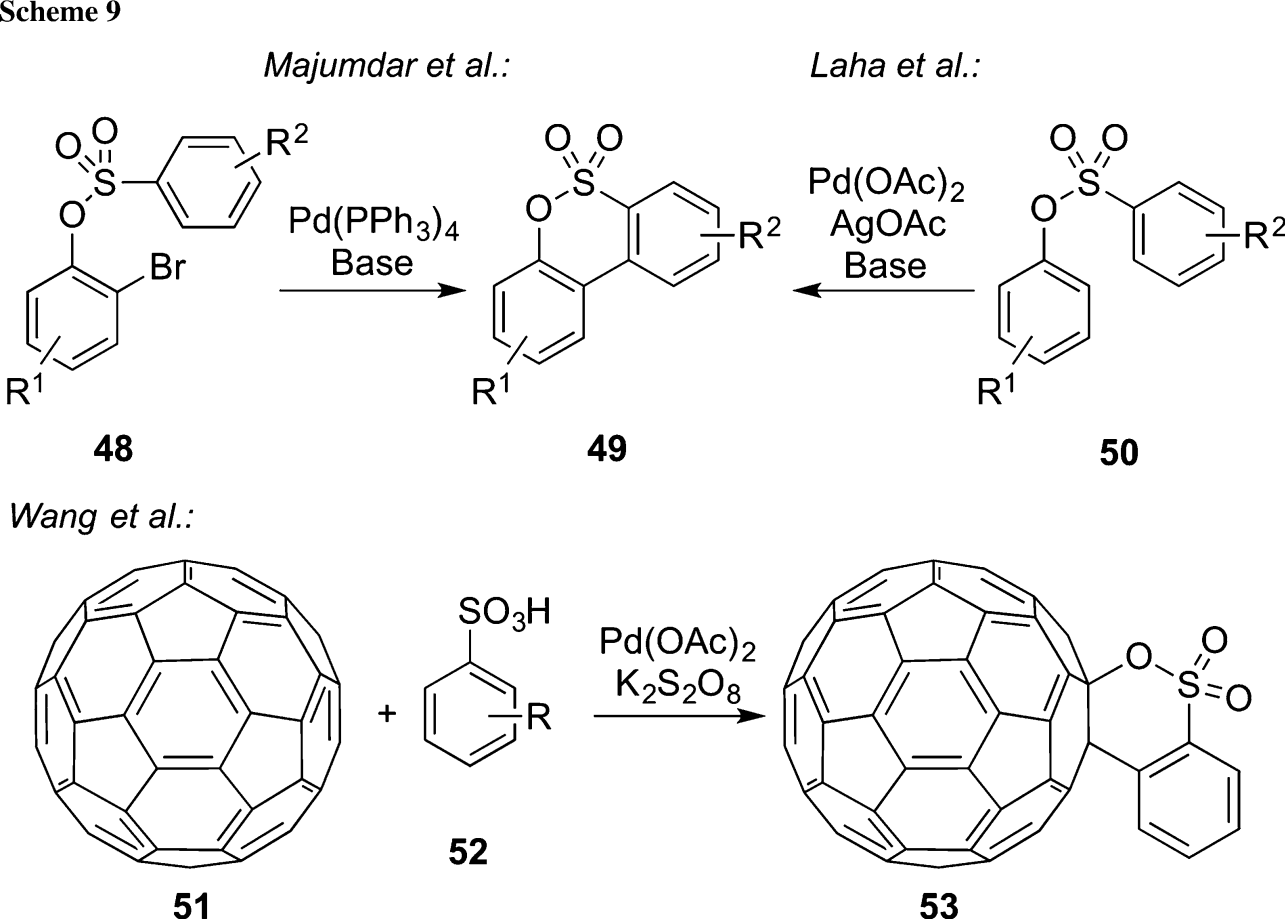

## Radical cyclizations

Cyclization reactions that proceed via radical species have for a long time attracted synthesis-oriented chemists and numerous highly spectacular examples for the construction of complex cyclic molecules starting from acyclic radical precursors have been reported in the past [75, 76]. In the early 1990s, Motherwell and co-workers reported the rearrangement of homopropargyl arylsulfonates **54** under radical conditions (Bu_3_SnH and AIBN) to give the δ-sultones **55** in a so far unprecedented manner (Scheme 10) [77–79]. This unique reaction was assumed to proceed via a radical *ipso*-substitution and subsequent rearrangement and was also subject to more detailed mechanistic studies substantiating this mechanistic proposal [77–80]. An interesting observation was made when submitting the iodine-containing sulfonate **57** to these radical conditions [81] (Scheme 10, lower reaction). Hereby, the direct cyclization products **49** as well as the *ipso*-substitution products **58** were obtained in varying ratios, strongly depending on the electronic nature and the location of the ring-substituent R.

**Figure:**
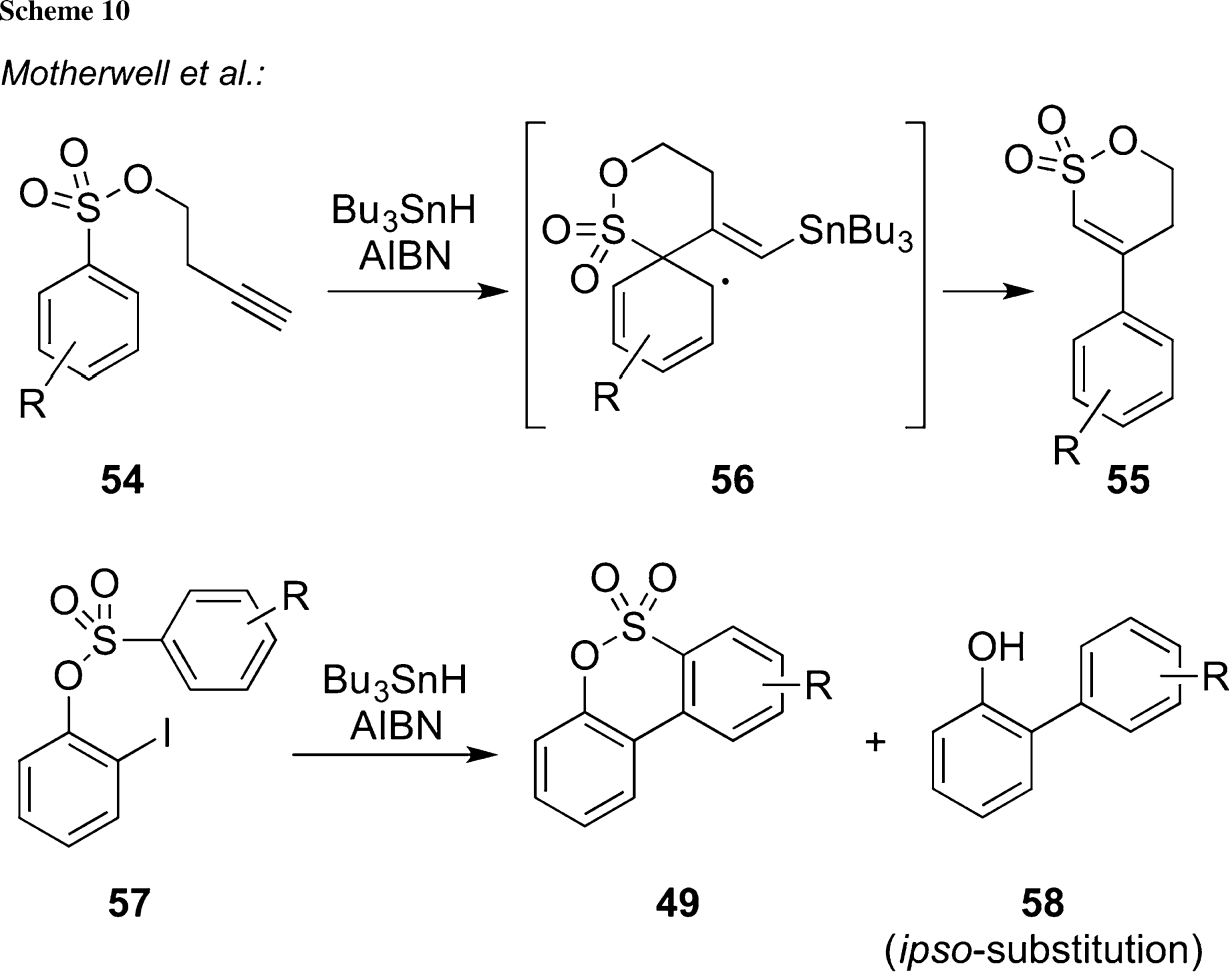

Another very interesting radical cyclization approach of sulfonic acid esters and amides **59** was recently reported by Tang, Shi, and co-workers [82]. By reacting compounds **59** with Togni’s electrophilic CF_3_-transfer reagent **60** under copper-catalysis, the trifluoromethylated δ-sultones/sultames **61** can be obtained in high yields and with a broad substrate scope (Scheme 11, upper reaction). It should be noted that the authors mainly used amides **59** (giving the sultames) but also proved the feasibility of this concept for a small variety of sulfonates to access the corresponding sultones **61**. Carefully chosen control experiments clearly support a radical mechanism for this transformation [82]. Very recently, Alcaide, Almendros, and co-workers developed a metal free radical cyclization strategy to access highly functionalized δ-sultones **64** starting from the radical precursor **62** and TEMPO (**63**) in the presence of trifluoroacetic acid [83]. Noteworthy, a variety of highly functionalized starting materials **62** (with rather complex R-groups) were obtained in a very elegant fashion from allene precursors and the interested reader is kindly referred to the original paper for more details concerning this interesting procedure [83].

**Figure:**
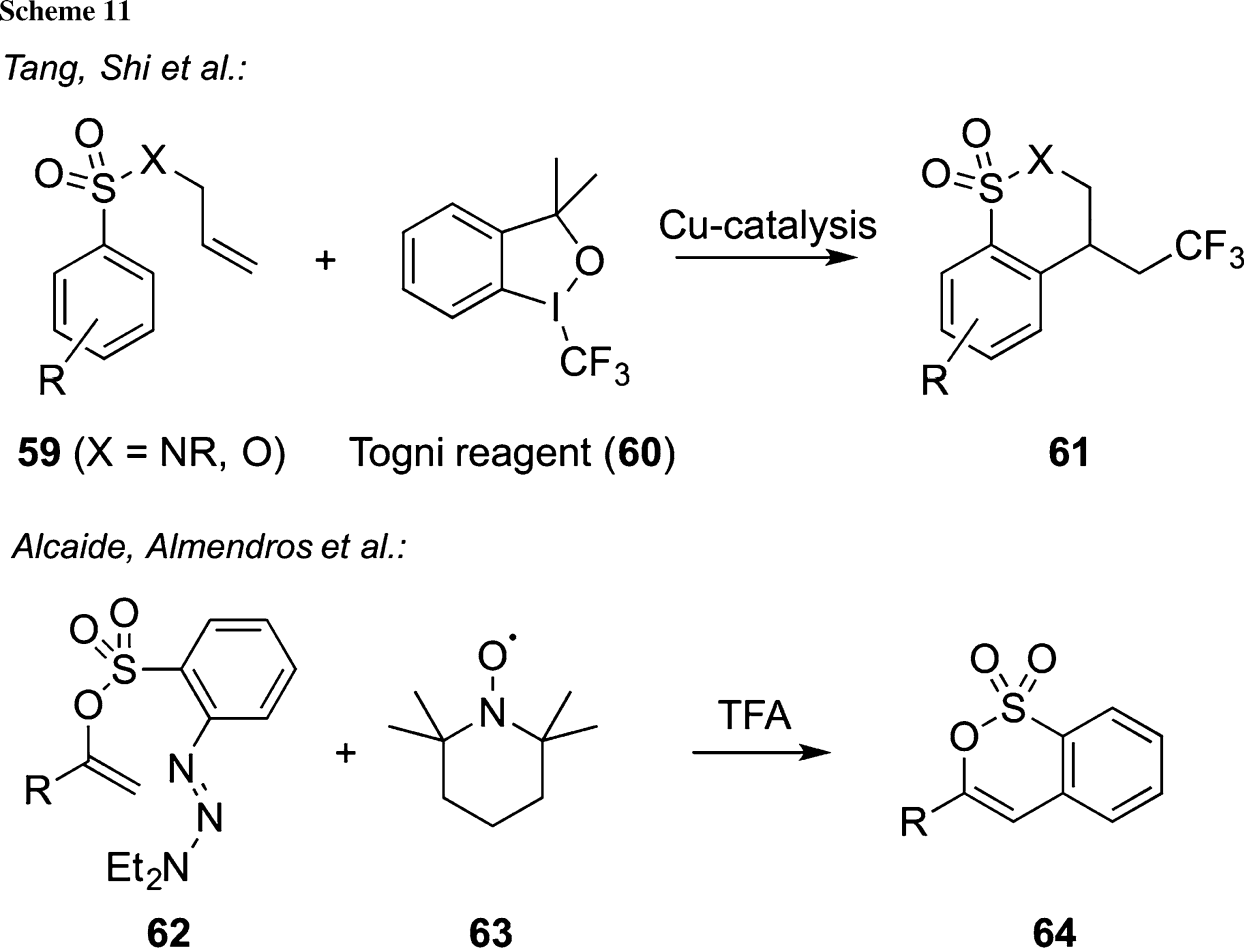

## Photoredox catalysis

Besides using stoichiometric oxidants/reductants or employing carefully functionalized precursors for (radical) cyclization reactions, such as those shown above, the use of photoredox catalysts to facilitate cyclizations under photochemical conditions has emerged as a very powerful tool. Interestingly, despite the longstanding history of photochemical methods, it has only been over the course of the last decade that a broad variety of new and generally applicable methods have been introduced and that these methods are more routinely used also in complex target-oriented syntheses (for two recent reviews and further details, please see [84, 85]). One particularly powerful application of photoredox catalysis is to carry out cross-coupling-type reactions. For example, Barriault et al. recently reported the use of dimeric gold complexes to carry out intramolecular cyclization reactions of bromide-containing tethered alkenes, alkynes, and aromatic compounds under photochemical conditions to give five- and six-membered carbo- and heterocycles [86]. Hereby, they also showed that this protocol can be successfully applied to the cyclization of sulfonates under UVA or sunlight irradiation (Scheme 12).

**Figure:**
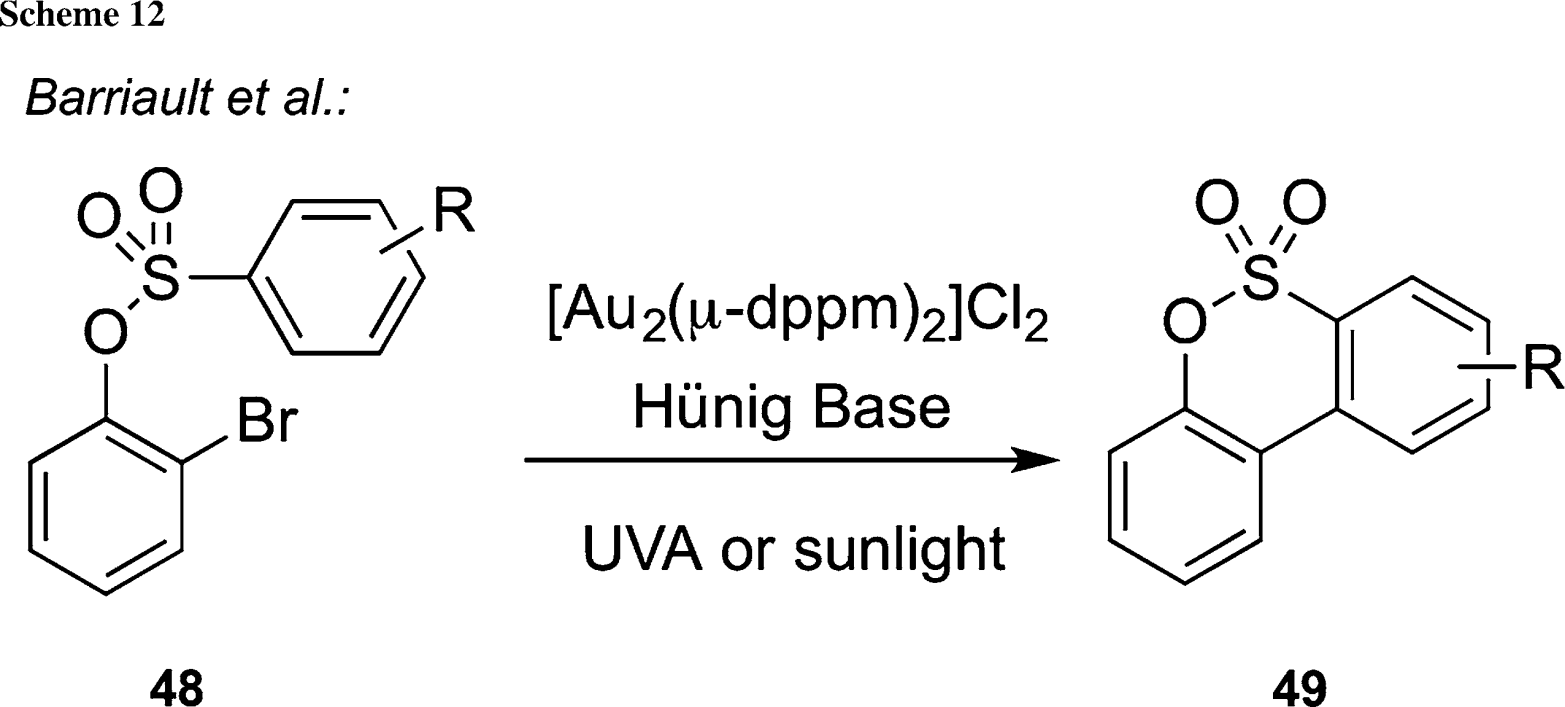

Very recently, Reiser’s group developed an interesting procedure for the synthesis of trifluoromethylated sultones starting from simple alkenols **65** under photoredox catalysis (Scheme 13) [87]. By reacting a variety of differently substituted compounds **65** with CF_3_SO_2_Cl in the presence of base and the photoredox catalyst **66** (dap = 2,9-di(*p*-anisyl)-1,10-phenanthroline) under irradiation with green light (530 nm LED), the trifluoromethylated δ-sultones **67** can be obtained in good-to-excellent yields, highlighting once again the potential of photoredox catalysis to access valuable target molecules in a highly efficient manner.

**Figure:**
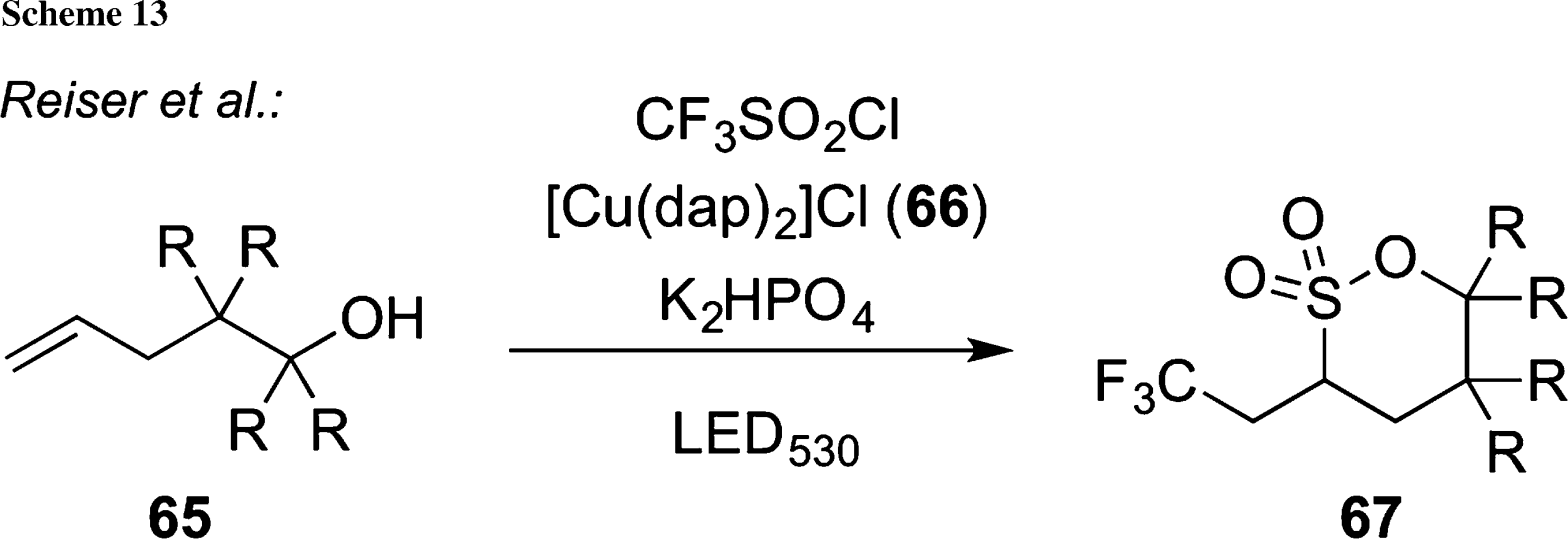

## Nucleophilic addition reactions

A broad variety of enolate-type nucleophilic addition reactions to access differently ring-sized sultones have been reported in the past [12, 13, 88–95], either via cyclization/addition of sulfonate-based carbanions to an adjacent electrophilic position (e.g., an ester or an aldehyde) or by adding enolates to activated electrophilic sulfuric acid species. In 1972 already, Timoney et al. carried out the base-mediated cyclization of mesylates **68** to access the bicyclic δ-sultones **69**, albeit in low yield [88]. This method was later on significantly improved by Arava’s group, who also demonstrated the use of compounds **69** as intermediates for further manipulations [89]. In an analogous fashion, the intramolecular aldol-type cyclization of ortho-formyl substituted O-mesylated phenol derivatives **70** has been successfully employed by several groups to access the valuable δ-sultones **71** (Scheme 14), which can then serve as building blocks for a variety of further transformations and as intermediates en route to potentially biologically active molecules [90–92]. One interesting example highlighting the potential of these cyclization approaches was reported in 2014 [12], where a broad variety of the potential anti BVDV (bovine viral diarrhea virus) active bicyclic δ-sultones **74** was access in a straightforward manner starting from lactones **73** under the basic conditions.

**Figure:**
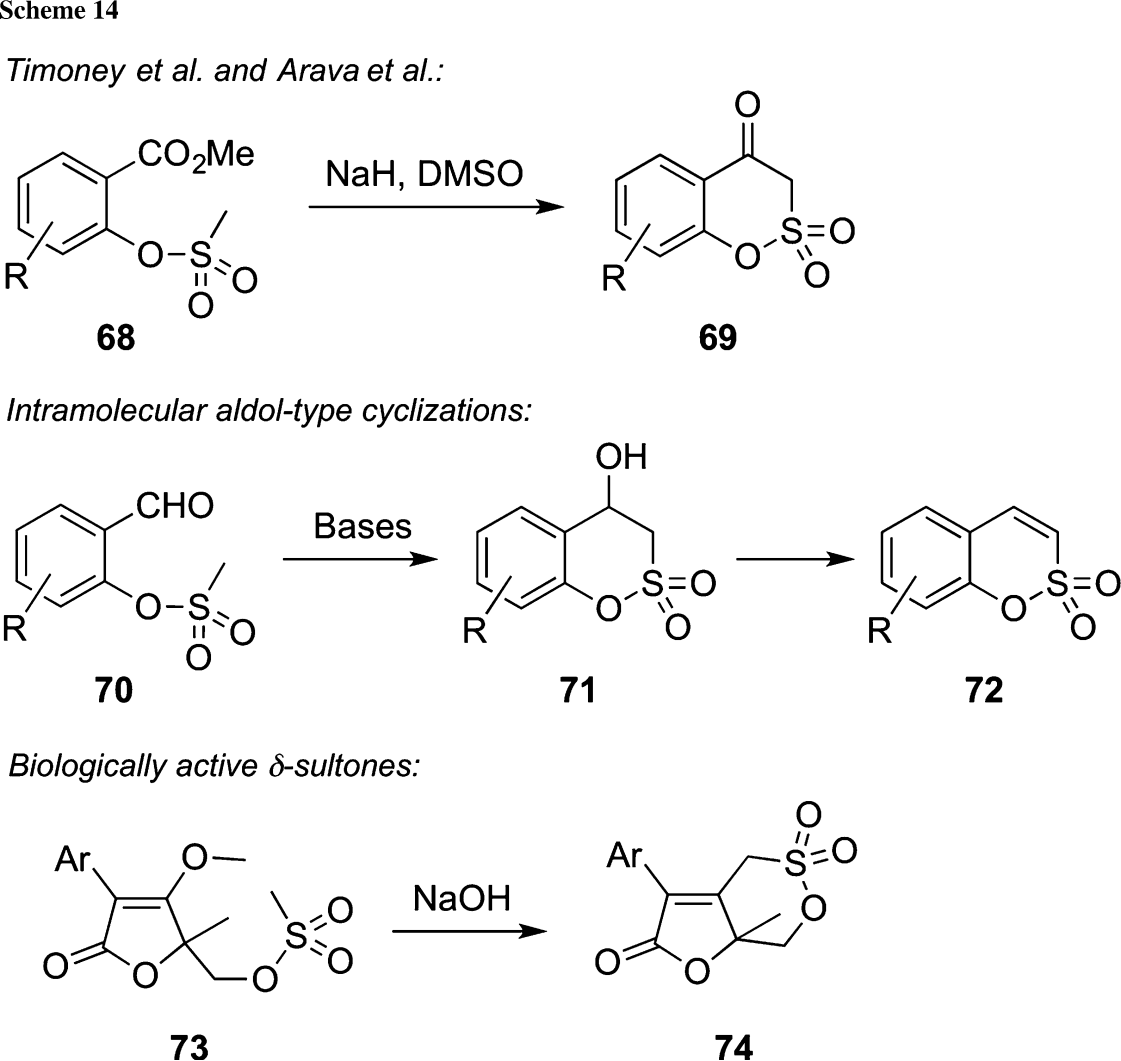

Some other very interesting nucleophilic addition-based strategies based on the use of simple sulfonates **75** were reported recently [93–95]. In 2012, Ghandi’s group reported an impressive protocol to access the highly functionalized polycyclic sultones **76** upon reacting **75** with different hydroxylamines [93]. Hereby, the primary addition products are supposed to undergo an immediate 1,3-dipolar cycloaddition reaction, as outlined in Scheme 15, resulting in a very efficient and also highly diastereoselective reaction. In an analogous manner, compounds **75** also served as starting materials for Knoevenagel/hetero-Diels–Alder reactions with either nucleophile **77** or **79** to obtain the highly functionalized pentacyclic δ-sultones **78** and **80** with high diastereoselectivities (Scheme 15, lower reaction) [94, 95].

**Figure:**
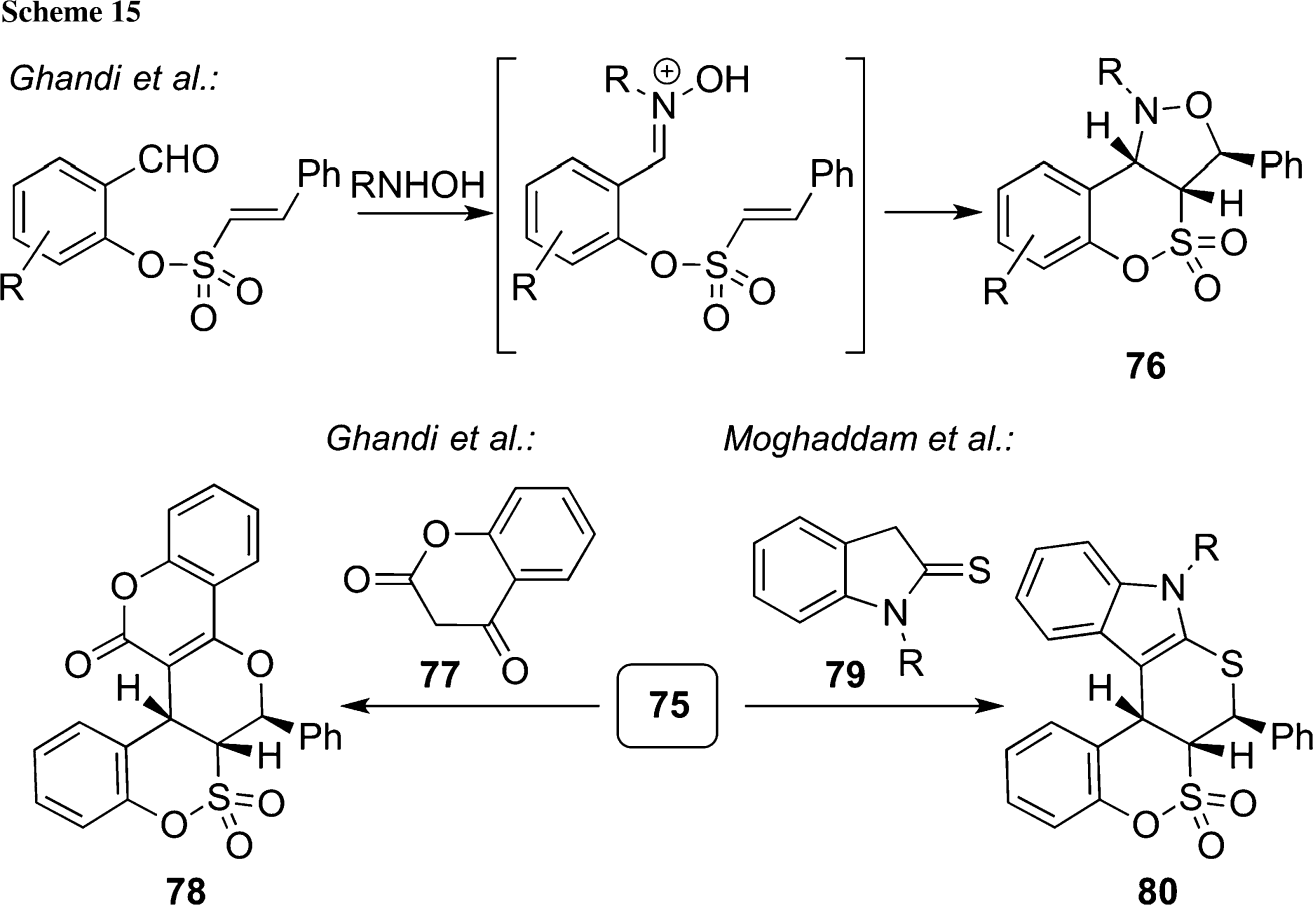

As mentioned in the introduction of this chapter, besides cyclizations of sulfonate carbanionic species, also addition reactions of other nucleophiles, such as, e.g., enolates to activated sulfuric acid species, have been successfully utilized for δ-sultone syntheses [13, 96]. In 2015, the groups of Wang and Lei reported the direct conversion of the highly functionalized conjugated enol **81** to the δ-sultone **82** upon treatment with acetic anhydride and sulfuric acid (Scheme 16, upper reaction). It was proposed that the reaction proceeds via a highly reactive mixed sulfuric acid–acetic acid anhydride which is attacked by the nucleophilic α-carbon of the enol species [13]. Compound **82** was then also subjected to a detailed biological testing and showed some interesting properties which may lead to more detailed future studies.

**Figure:**
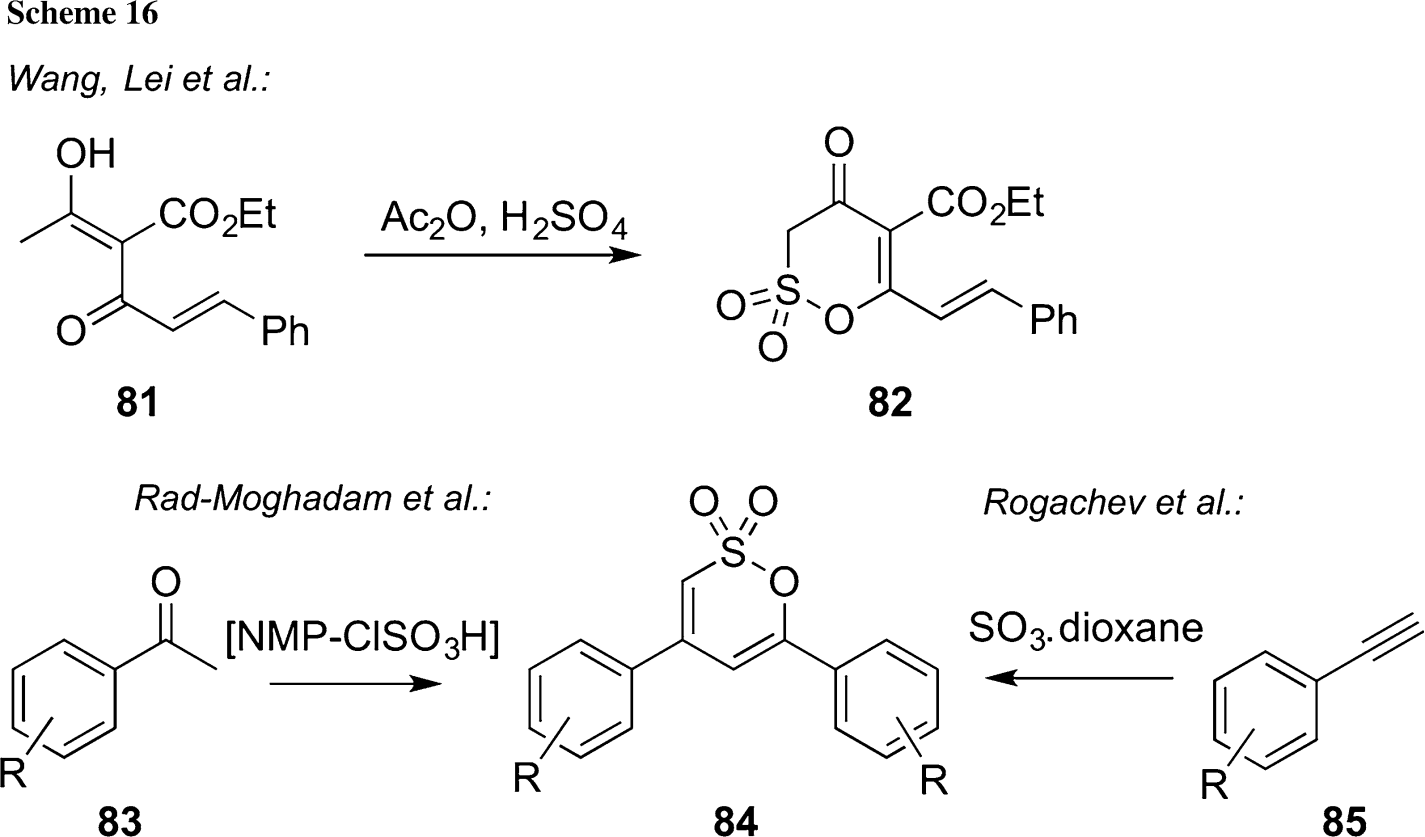

Very recently, Rad-Moghadam’s group reported the use of the easily accessible ionic liquid [NMP-ClSO_3_H] (NMP = *N*-methyl-2-pyrrolidone) as a sulfonating agent which allows for the straightforward syntheses of δ-sultones **84** starting from simple acetophenone derivatives **83** [96]. This protocol also resembles a slightly older report by Rogachev and co-workers who succeeded in carrying out the direct formation of sultones **84** from phenylacetylene derivatives **85** upon treatment with SO_3_ dioxane complex [97].

## Conclusion

The development of efficient and functional group tolerant synthesis methods to access δ-sultones has become an important task over the last decades. A variety of complementary strategies have been introduced so far, making these important targets accessible from different easily available starting materials. Importantly, because of the versatility of δ-sultones for further transformations, the recently developed synthesis approaches, therefore, provide powerful tools which are often worth being considered in the retrosynthetic planning of complex molecule total synthesis, which was impressively shown in different case studies already. We hope that we could provide an illustrative overview of this fascinating field and we are confident that these heterocycles will remain being an important source of motivation and inspiration for the development of new synthesis and catalysis methods.
